# Targeting autoimmune mechanisms by precision medicine in Myasthenia Gravis

**DOI:** 10.3389/fimmu.2024.1404191

**Published:** 2024-06-06

**Authors:** Paola Cavalcante, Renato Mantegazza, Carlo Antozzi

**Affiliations:** ^1^ Neurology 4 – Neuroimmunology and Neuromuscular Diseases Unit, Fondazione IRCCS Istituto Neurologico Carlo Besta, Milan, Italy; ^2^ Immunotherapy and Apheresis Unit, Fondazione IRCCS Istituto Neurologico Carlo Besta, Milan, Italy

**Keywords:** Myasthenia Gravis, autoimmunity, B cells, neonatal Fc receptor, complement system, precision medicine

## Abstract

Myasthenia Gravis (MG) is a chronic disabling autoimmune disease caused by autoantibodies to the neuromuscular junction (NMJ), characterized clinically by fluctuating weakness and early fatigability of ocular, skeletal and bulbar muscles. Despite being commonly considered a prototypic autoimmune disorder, MG is a complex and heterogeneous condition, presenting with variable clinical phenotypes, likely due to distinct pathophysiological settings related with different immunoreactivities, symptoms’ distribution, disease severity, age at onset, thymic histopathology and response to therapies. Current treatment of MG based on international consensus guidelines allows to effectively control symptoms, but most patients do not reach complete stable remission and require life-long immunosuppressive (IS) therapies. Moreover, a proportion of them is refractory to conventional IS treatment, highlighting the need for more specific and tailored strategies. Precision medicine is a new frontier of medicine that promises to greatly increase therapeutic success in several diseases, including autoimmune conditions. In MG, B cell activation, antibody recycling and NMJ damage by the complement system are crucial mechanisms, and their targeting by innovative biological drugs has been proven to be effective and safe in clinical trials. The switch from conventional IS to novel precision medicine approaches based on these drugs could prospectively and significantly improve MG care. In this review, we provide an overview of key immunopathogenetic processes underlying MG, and discuss on emerging biological drugs targeting them. We also discuss on future direction of research to address the need for patients’ stratification in endotypes according with genetic and molecular biomarkers for successful clinical decision making within precision medicine workflow.

## 1 Introduction

Myasthenia gravis (MG) is a chronic T cell-dependent, B cell-mediated autoimmune disease caused by autoantibodies against proteins of the neuromuscular junction (NMJ) that impair neuromuscular transmission leading to disabling muscle weakness and fatigability (1).

Despite it is commonly considered a prototypic antibody-mediated autoimmune condition, MG is a complex and heterogeneous disorder, characterized by fluctuating symptoms, unpredictable disease course and wide clinical and immunopathological variability, including different autoantibody specificities, age at onset, distribution of muscle weakness, thymic histopathology and clinical response to therapies (1, 2). This variability likely reflects distinct pathophysiological states and differences in the mechanisms underlying the production of autoantibodies and their pathogenic effects, understanding of which is the prerequisite for the development of tailored and more specific approaches to treat different subgroups of MG patients.

Currently, MG treatment is mainly based on symptomatic agents and non-selective immunosuppression with corticosteroids and/or non-steroidal immunosuppressants, able to control symptoms but source of side effects and rarely leading to complete stable remission (3, 4),. Moreover, about 10% of MG patients (~10%) are considered refractory to immunosuppressive (IS) drugs (4, 5). They represent a subgroup of patients treated with multiple drugs for long periods of time with considerable burden due to disability caused by uncontrolled MG and long-term immunosuppression (6). In this regard, the use of more specific therapeutic strategies is an urgent medical need for these patients. Biological drugs targeting disease effector mechanisms (3, 4, 7) are promising for the treatment of refractory MG patients as emerged from controlled studies. The introduction of these innovative drugs in the clinical practice could prospectively have a tremendous impact on therapeutic success in MG, especially when tailored treatments take into account the biological diversity among disease subgroups. Moreover, the introduction of innovative compounds early in the course of the disease could considerably reduce the risk of becoming refractory and limit the use of corticosteroids.

In this review, we provide an overview of the pathophysiological mechanisms targeted by innovative drugs in MG, and summarize data on their effectiveness in MG treatment. We also speculate on future directions of research to achieve precision medicine in MG.

## 2 MG heterogeneity, clinical subgroups and treatments

MG is a heterogeneous disease in which the variability allows patients’ stratification in distinct disease subgroups according to the following features: i) autoantibody status; ii) distribution of weakness; iii) age at the disease onset; and iv) histopathology of thymus (1, 2). Stratification of MG patients according to clinical and immunological features such as autoantibody specificity is now mandatory due to the different targets of innovative drugs, and therefore relevant to treatment decisions and outcomes, representing a first step towards personalization of therapies.

In most (80–85%) MG patients, the autoantibody target is the acetylcholine receptor (AChR) and its clustering in the post-synaptic membrane of NMJ is essential for muscle contraction. Less frequently, autoantibodies are directed to the muscle-specific tyrosine kinase (MuSK), or to the lipoprotein-related protein 4 (LRP4), two proteins implicated in AChR clustering. A very small proportion of patients have no specific autoantibodies (seronegative MG, or triple negative MG) and in these patients the neurophysiological confirmation of the diagnosis is mandatory (1, 2). Anti-AChR, -MuSK and -LRP4 autoantibodies have proven pathogenicity, since they are able to induce end-plate alterations and impairment of neuromuscular transmission (8–10).

Ocular symptoms are often observed at onset, then shifting to a generalized form (gMG) usually within two years, which involves skeletal and bulbar muscles (11).

Commonly, MG patients are stratified according to age at onset in patients with early- (EOMG, < 50 years) or late- (LOMG, > 50 years) onset disease (12). EOMG is more common in women than in men, and frequently shows thymic follicular hyperplasia, which is present in most (~ 80%) AChR-positive patients (1, 2, 13). LOMG is more frequent in males, who often have a normally involuted thymus and antibodies to other muscle proteins, mainly titin or the ryanodine receptor (RyR) (1, 14). An increase in MG incidence in the elderly population, particularly in subjects over 65 years, has been reported in recent years, likely due to improved diagnosis; these patients are mostly patients with anti-AChR antibodies and without thymoma (14, 15).

Thymomas are detected in approximately 10–15% of MG patients. Thymoma-associated MG can occur at any age and is characterized by the presence of anti-AChR antibodies and generalized disease in almost all patients, who frequently have additional autoantibodies directed to titin and RyR that are useful as biomarkers to diagnose thymoma in patients younger than 50 years (1, 16).

Disease severity is variable among patients. MuSK-MG and MG associated with thymoma have a higher risk of a severe clinical course compared to the other clinical subgroups (1, 2).

Several variables need to be taken into account for making clinical decisions in MG, including weakness severity, muscles involved, thymus pathology, autoantibody specificity and patient comorbidities.

International and national treatment guidelines are available for MG treatment (3, 17). The current therapeutic algorithm includes the following steps: 1) symptomatic therapy with cholinesterase inhibitors (first-line treatment); 2) corticosteroids, alone or combined with other IS agents (second-line treatment); 3) thymectomy in selected patients (young onset AChR-positive patients, or thymoma), and 4) plasmapheresis/immunoglobulins for acute exacerbations (2–4). The prognosis for MG patients has greatly improved over the past half century, with substantial reductions in mortality and morbidity. Nevertheless, inter-individual variability in drug effectiveness, adverse events related to conventional treatments, co-morbidities limiting corticosteroid usage and treatment refractoriness (5, 6) are a challenge to clinicians, requiring the development of more specific and better tolerated therapeutic approaches.

**Figure 1:**
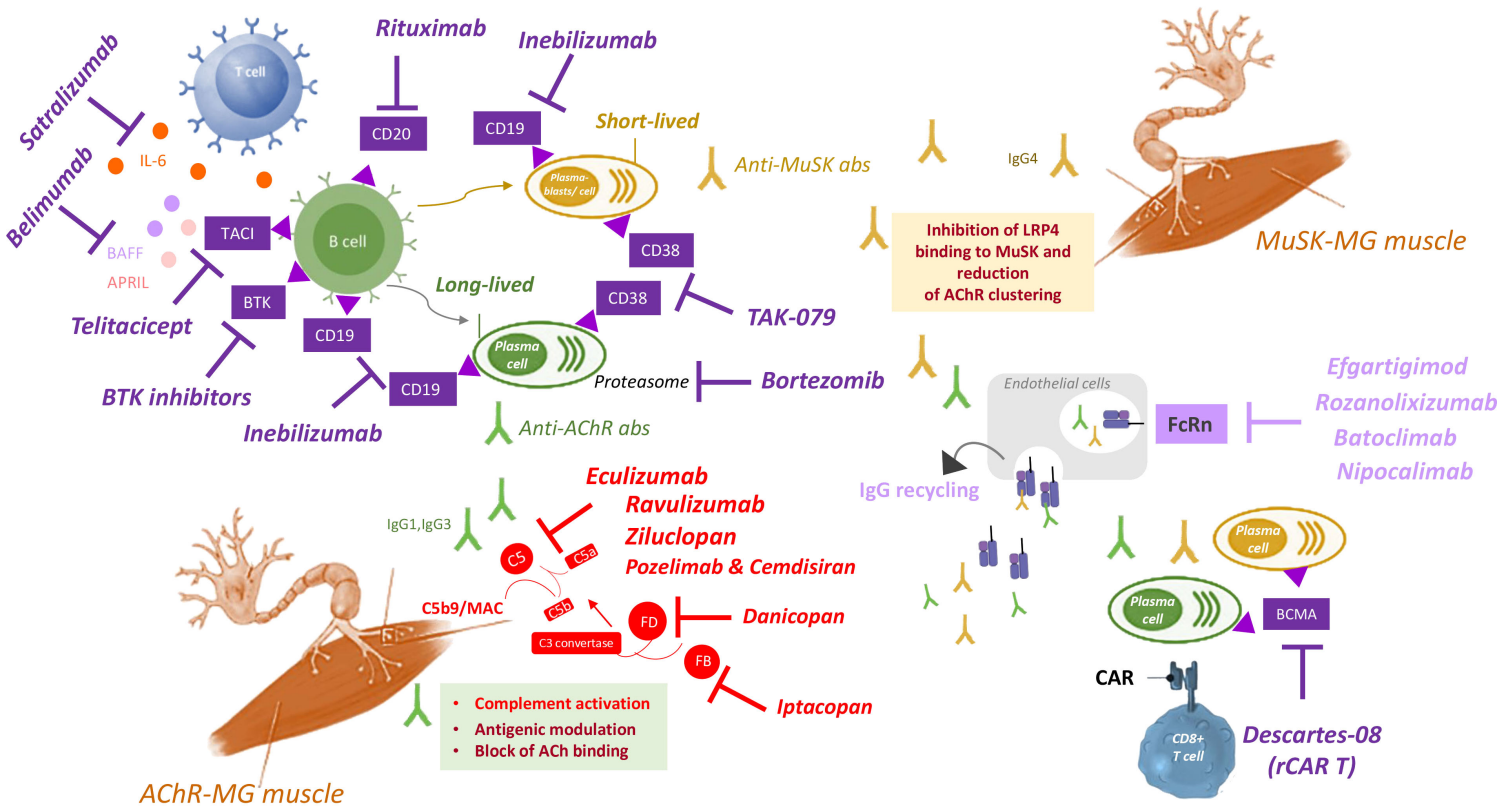
Summary of targeted therapies for MG treatment. Autoantibodies to AChR (green) produced by long-lived plasma cells (green) impair NMJ by the following mechanisms (green box): i) complement activation and MAC formation, ii) antigenic modulation, or iii) block of the ACh binding to the receptor. Autoantibodies to MuSK (yellow) produced by short-lived plasma blasts (yellow) inhibit LRP4 binding to MuSK, thus compromising AChR clustering (yellow box). Several biological drugs (purple, upper left quadrant) are available to directly target B or plasma cells, or to block B cell survival and activation, including: i) rituximab, that targets CD20 expressed on B cells; ii) inebilizumab, that targets CD19 expressed on B cells, plasmablasts and plasma cells; iii) BTK inhibitors, that target BTK expressed in B cells; iv) bortezomib, that inhibits proteasome to deplete plasma cells; v) TAK-079, that targets CD38 expressed on plasmablasts and plasma cells; vi) belimumab, that targets the B cell survival factor BAFF (also called Blys); vii) telitacicept, that targets the TACI receptor to inhibit the B cell survival factors BAFF and APRIL; viii) satralizumab, that targets IL-6 to inhibit B cell activation and differentiation. CAR T cells engineered with RNA (rCAR-T) has been recently developed as therapeutic strategy to block plasma cells, by specifically targeting BCMA expressed in these cells (lower right quadrant). Drugs targeting the FcRn (light purple, middle right quadrant) inhibit IgG recycling, thus reducing autoantibody levels. They include: efgartigimod, rozanolixizumab, batoclimab and nipocalimab. Drugs targeting the complement system (red, lower left quadrant) are effective to treat the disease in patients with anti-AChR antibodies, by inhibiting complement activation. They include eculizumab, ravulizumab, zilucoplan, and the combination of pozelimab and cemdisiran, all able to target C5, thus inhibiting its cleavage in C5a and C5b, and hence MAC (C5b9) formation. Additional targets of anti-complement drugs are Factor D, targeted by danicopan, and Factor B, targeted by Iptacopan, two complement factors implicated in the formation (factor D) and amplification (factor B) of the process that leads to C3 convertase formation, that is essential for the formation of C5 convertase and hence for C5 cleavage and subsequent MAC formation. Abs: antibodies; ACh: acetylcholine; AChR: acetylcholine receptor; APRIL: a proliferation-inducing ligand, also known as tumor necrosis factor ligand superfamily member 13 (TNFSF13); BAFF: B-cell activating factor, also known as B lymphocyte stimulator (Blys); BCMA: B-cell maturation antigen; BTK: Bruton’s tyrosine kinase; CAR: chimeric antigen receptor; FcRn: neonatal Fc receptor; IgG: immunoglobulin G; IL-6: interleukin 6; LRP4: low density lipoprotein receptor-related protein 4; MAC: membrane attack complex; MuSK: muscle-specific kinase receptor; NMJ: neuromuscular junction; rCAR T: CAR T cells engineered with RNA; TACI: transmembrane activator and CAML interactor.

In recent years, several biological drugs have been developed for MG treatment (4, 7, 18), namely biological compounds targeting B cells, the neonatal Fc receptor (FcRn) and the complement system (**Tables 1**–**3**, **Figure 1**), representing different tools able to interfere with key steps of the autoimmune process in MG (18). They proved to be effective for the treatment of MG patients/patients’ subgroups, in line with the crucial role of their biological targets in the disease pathogenesis, as discussed in the following paragraphs.

**Table 1:** B cell-targeted biological drugs in myasthenia gravis.

Biological drug	Drug molecule	Target	Drug function	Clinical studies	Treatment outcomes
Rituximab (RTX)	Chimeric murine-human IgG1 mab	CD20	Depletion of CD20+ B cells by apoptosis induction	BeatMG – Phase 2 study in AChR+ patients with mid-to-moderate gMG	No significant steroid-sparing effect^59^
				Long-term follow-up (31 months) of RTX-treated AChR+ and MuSK+ MG patients	Autoab decrease, remission/minimal manifestations in MuSK-MG, but not in AChR-MG, patients^60^
				RINOMAX – Clinical study in new-onset AChR+ patients with gMG	Increased probability of minimal manifestations and reduction of the need of rescue medications^63^
				REFINE – new trial on RTX efficacy in AChR+ MG patients	Ongoing trial (NCT05868837), expected to be completed in 2025
Inebilizumab	Human mab	CD19	Cytotoxicity and depletion of CD19+ B cells	MINT – new trial in AChR+ and MuSK+ gMG patients	Ongoing trial (NCT04524273), expected to be completed in 2029
Bortezomib	Pyrazine-containing small molecule	26S proteasome	Proteasome inhibition and plasma cell depletion	TAVAB – Phase 2 study in treatment-refractory patients with autoimmune diseases, including AChR+ MG patients	No results published yet (NCT02102594)^69^
TAK-079	Human mab	CD38	Cellular cytotoxicity, lysis and depletion of CD38-expressing cells, including plasmablasts and plasma cells	Phase 2 study in AChR+ and MuSK+ gMG patients	No publication available (NCT04159805)
Descartes - 08	Autologous CAR T cells engineered with RNA to target BCMA	BCMA	Inhibition of plasma cells	MG-001 – OLE phase 1b/2a study in seropositive (AChR+, MuSK+ or LRP4+) gMG patients	Decrease of MG-ADL, MGC, QMG, and MG-QoL-15r scores at up to 9 months of follow-up^71^
Belimumab	Human mab	BAFF	Blocking of BAFF, inhibition of its interaction with BAFF-R, BCMA and TACI receptors and reduction of B cell survival	BEL115123 – Phase 2 study on gMG patients all AChR+, except 2 MuSK+, with mild-moderate disease	No significant QMG score change after 24 weeks^74^
Telitacicept	Fusion protein	TACI	Inhibition of BAFF and APRIL binding to the TACI receptor on B cells and reduced B cell survival	Phase III Study in AChR+ or MuSK+ patients with gMG	Ongoing trial (NCT05737160), expected to be completed in 2027
Satralizumab	Humanised mab	IL-6	Inhibition of IL-6 signaling	LUMINESCE – Phase 3 study in seropositive (AChR+, MuSK+, LRP4+) gMG patients	Modest improvement in MG-ADL and QMG scores from baseline to week 24 in AChR-MG patients (NCT04963270, Abstract, AAN 2024^a^)

AChR, acetylcholine receptor; Autoab, autoantibodies; BAFF, B lymphocyte stimulator; BAFF-R, BAFF receptor; BCMA, B-cell maturation antigen; CAR T, chimeric antigen receptor T cells; gMG, generalized myasthenia gravis; IL-6, interleukin-6; LRP4, lipoprotein-related protein 4; Mab, monoclonal antibody; MG-ADL, MG-Activities of Daily Living score; MGC, MG Composite score; MG-QoL15r, MG Quality of Life 15-item Scale; MuSK, muscle-specific kinase receptor; OLE, open label extension; QMG, quantitative myasthenia gravis score; TACI, transmembrane activator and CAML interactor.

a AAN: American Academy of Neurology, annual meeting 2024, abstract from: https://medically.roche.com/global/en/neuroscience/aan-2024/medical-material/AAN-2024-poster-habib-LUMINESCE-a-phase-3-study-of-satralizumab-pdf.html (access on 13 may 2024)

**Table 2:** Biological drugs targeting FcRn in myasthenia gravis.

Biological drug	Drug molecule	Target	Drug function	Clinical studies	Treatment outcomes
Efgartigimod (ARGX-113)	Humanized IgG1-derived Fc fragment	FcRn	Binding of FcRn with high affinity, competition with endogenous IgG, inhibition of IgG recycling and reduction of IgG levels, including autoabs	ADAPT – Phase 3 study in AChR+, MuSK+ and double (AChR/MuSK)-seronegative patients with gMG	Reduction of IgG levels in AChR+ and seronegative patients. Sustained improvements of MG-ADL, QMG, MGC, and MG-QoL15r scores in AChR+ and seronegative patients^92^
				Retrospective study in AChR+ patients with gMG	MG-ADL reduction after the first cycle; minimal symptom expression in 25% of patients after 1 and in 25% after 2 cycles with Sustained benefits after cycle 2^93^
				Study on the long-term effect (14 months) of the drug in AChR+, MuSK+, LRP4+, and triple negative gMG patients	MG-ADL, MGC and QMG improvement at the end of each cycle. No patient hospitalization during the treatment compared to the year before treatment^94^
				Study on drug efficacy in seropositive and seronegative gMG patients	MG-ADL, MGC and MG-QoL15r improvement in 34/36 patients after the first and subsequent cycles ^95^
				ADAPT – OLE study (up to 3-year extension)	Confirmation of long-term efgartigimod safety, tolerability, and efficacy^96^
Rozanolixizumab (UCB7665)	Human IgG4 mab	FcRn	Binding of FcRn, competition with endogenous IgG, inhibition of IgG recycling and reduction of IgG levels, including autoabs	MycarinG – Phase 3 study in AChR+ and MuSK+ patients with gMG	Significant MG-ADL reductions at day 43; improvements in MG-ADL, MGC, QMG and Myasthenia Gravis Symptoms PRO as early as day 8 up to day 99^97^
Nipocalimab (M281)	Human IgG1 mab	FcRn	Binding of FcRn, competition with endogenous IgG, inhibition of IgG recycling and reduction of IgG levels, including autoabs	Vivacity-MG – Phase 2 study in moderate-to-severe AChR+ and MuSK+ patients with gMG and inadequate response to stable standard-of-care	Rapid improvement (within 2 weeks) in MG‐ADL across 4 dosing arms, and reduction in total IgG and anti-AChR abs^99^
				Phase 3 study in seropositive patients with gMG	Ongoing trial (NCT04951622), expected to be completed in 2026
Batoclimab	Human mab	FcRn	Binding of FcRn, competition with endogenous IgG, inhibition of IgG recycling and IgG reduction, including autoabs	Phase 2 study with an OLE in AChR+ patients with gMG	Reduction in total IgG across IgG subclasses and anti-AChR abs, but no significant changes in G-ADL, QMG, MGC, and MG-QoL15r scores^100^
				Phase 3 study in AChR+ and MuSK+ patients with gMG	Significant increase in the rate of sustained MG-ADL improvement ^101^
				Phase 3 study in gMG patients	Ongoing trial (NCT05403541), expected to be completed in 2025

AChR, acetylcholine receptor; Autoab, autoantibodies; FcRn, neonatal Fc receptor; gMG, generalized myasthenia gravis; LRP4, lipoprotein-related protein 4; Mab, monoclonal antibody; MG-ADL, MG-Activities of Daily Living score; MGC, MG Composite score; MG-QoL15r, MG Quality of Life 15-item Scale – Revised score; MuSK, muscle-specific kinase receptor; OLE, open-label extension; QMG, quantitative myasthenia gravis score.

**Table 3:** Biological drugs targeting the complement system in myasthenia gravis.

Biological drug	Drug molecule	Target	Drug function	Clinical studies	Treatment outcomes
Eculizumab	Humanized mab	C5	Inhibition of C5 cleavage into C5a and C5b and of MAC formation with reduction of the complement-mediated damage	REGAIN – Phase 3 study in treatment-refractory AChR+ patient with gMG and its OLE study	Significant MG-ADL and QMG reduction; rapid (i.e. by weak 1) and sustained (i.e. at least 130 weeks) improvement in ocular, bulbar, respiratory, and limb muscle strength and in daily activities^125-127^
Ravulizumab	Humanized mab	C5	Inhibition of C5 cleavage into C5a and C5b and of MAC formation with reduction of the complement-mediated damage	CHAMPION-MG – Phase 3 study in AChR+ patients with gMG and its OLE study	Significant MG-ADL and QMG reduction within 2 weeks and maintained through 60 weeks^128,129^
Ziluclopan	Macrocyclic peptide	C5	Allosteric inhibition of C5 cleavage and of MAC formation with reduction of the complement-mediated damage	RAISE – Phase 3 study in AChR+ patients with moderate to severe gMG	Significant reduction in MG-ADL score from baseline to week 12^130^
				RAISE-XT – OLE phase of RAISE	Ongoing trial, expect to be completed in 2026 (NCT04225871)
Pozelimab Cemdisiran	Pozelimab: human IgG4 (IgG4P) mab Cemdisiran: small interfering N-acetylgalactosamine-conjugated ribonucleic acid (siRNA)	C5	Pozelimab: inhibition of C5 cleavage and of MAC formation with reduction of the complement-mediated damage; Cemdisiran: Interference with the mRNA for C5, reduction of C5 expression and circulating levels	NIMBLE – Phase 3 study on the efficacy of a combined therapy based on the two drugs in AChR+ patients with gMG	Ongoing trial, expected to be completed in 2028 (NCT05070858)
Vemircopan	Oral inhibitor molecule	Factor D	Inhibition of C3 convertase formation and of MAC formation	Phase 2 study in AChR+ patients with gMG	Ongoing trial, expected to be completed in 2025 (NCT05218096)

AChR, acetylcholine receptor; gMG, generalized myasthenia gravis; Mab, monoclonal antibody; MAC, membrane attack complex; MG-ADL, MG-Activities of Daily Living score; QMG, quantitative myasthenia gravis score.

## 3 B cells: key players and therapeutic targets in MG

B cells are implicated in MG pathogenesis, since they produce the specific autoantibodies. Their contribution to MG has been widely investigated, and significant thymic and peripheral B cell dysfunctions in MG patients have been demonstrated (19).

B cells, as the major component of the humoral adaptive immune response, have always represented an attractive target for effective therapeutic intervention in autoimmune diseases, such as MG.

### 3.1 Role of B cells in MG pathogenesis

#### 3.1.1 B cells in MG thymus

The thymus is a site of relevant B cell abnormalities in AChR-MG patients. B cell infiltration and development of ectopic germinal centers (GC) forming follicles are histopathological features of thymic follicular hyperplasia, which characterizes the majority of these patients (13, 19). B cells from hyperplastic MG thymus express markers of activation, including CD71, 4F2, CD23, and B8.7, and display functional signs of activation (20).

Ectopic lymphoid neogenesis is a feature commonly found in inflamed target tissues of patients with several autoimmune conditions, such as rheumatoid arthritis (RA) synovia, multiple sclerosis (MS) meninges, Sjogren’s syndrome salivary gland, systemic lupus erythematosus (SLE) kidneys, and Hashimoto’s thyroiditis thyroid (21). The exact mechanisms leading to GC formation in these organs, and in MG thymus, is still unknown. GCs generally develop in the context of chronic inflammation. Indeed, the follicular hyperplastic MG thymus is characterized by a well-defined chronic inflammatory condition, and presents features of tertiary lymphoid organs, such as sustained over-expression of inflammatory cytokines and chemokines, GC development, and neoangiogenic processes (i.e. high endothelial venule and lymphatic vessel development) (22–25). The B cell-attracting chemokines CXCL13 and CCL21, key molecules over-expressed in follicular hyperplastic MG thymuses, contribute to the formation and maintenance of ectopic follicles by promoting active recruitment of peripheral B cells, as well as T cells, into the thymus (22–25). Peripheral B cells attracted by the two chemokines can be sensitized against AChR locally expressed in the thymus (i.e. thymic epithelial cells and myoid cells) (26–28). In this regard, there is evidence that the hyperplastic MG thymus contains plasma cells that are able to produce anti-AChR antibodies *in vitro* (29). Ectopic GCs are known to be the site of affinity maturation, clonal selection and differentiation of autoreactive B cells (19). In MG, ectopic GCs contain B cells undergoing antigen-driven clonal expansion, somatic hypermutation, and selection (30), indicating that they represent an immunological niche of autoreactive B cell differentiation and autoantibody production. Of interest, the GC number (i.e. degree of thymic hyperplasia) was found to correlate with autoantibody titers, and to decrease in MG patients undergoing corticosteroid treatment (31).

Pathogen infections are thought to play a role in chronic inflammation, type I interferon (IFN-I)-mediated anti-viral response and innate immune activation in hyperplastic MG thymuses (32, 33). IFN-β has been proven to play a major role in intra-thymic autosensitization to the AChR, since it is able to increase the expression of the AChR-α subunit by thymic epithelial cells, at the same time increasing the expression of CXCL13, CCL21 and BAFF (also known as B lymphocyte stimulator or BlyS), an important survival factor for B cells (34). Poliovirus-infected macrophages, and latently and litycally Epstein–Barr virus (EBV)-infected B cells and plasma cells, were detected in MG but not in normal control thymuses (32, 33), supporting the hypothesis that IFN-β over-expression and chronic inflammation might be triggered by persistent viruses in MG thymuses. Due to EBV unique ability to induce abnormal B cell activation, proliferation and survival (35), EBV persistence and reactivation observed in hyperplastic thymuses of AChR-MG patients represent an important source of B cell dysfunction, possibly contributing to the chronicity of the intra-thymic inflammatory autoimmune process. MG GCs were found to be EBV-enriched (33), similarly to GCs in other target organs of B cell-mediated autoimmune diseases, such as RA synovia and MS brain (36, 37), thus supporting EBV involvement in GC formation and B-cell dysregulation in different autoimmune conditions, including MG.

Polyclonality of thymic B cells was observed in MG patients (38), in line with widespread B cell activation by EBV. Of interest, thymus-associated antigen-experienced B cell clones were detected in the circulation of AChR-MG patients after thymectomy (39), the persistence of which correlated with reduced clinical response to surgery, thus indicating that autoreactive B cells can migrate to other immunological compartments and perpetuate autoimmunity in the periphery.

B cell abnormalities can be also observed in MG thymomas. Neoplastic tissue of MG patients’ thymomas is characterized by increased B cell infiltration compared to thymoma tissue from patients without MG (40). GCs encircled by high endothelial venules, that are potentially able to recruit peripheral B cells, were identified in the adjacent tissues of a high proportion of MG thymomas, and their number positively correlated with anti-AChR titers (41). Of note, EBV latency markers were found in thymoma-infiltrating B cells in MG but not in non-MG thymomas, thus supporting the virus contribution to intra-thymic GC formation and B cell-mediated autoimmunity also in thymoma-associated MG (40).

#### 3.1.2 B cells in MG peripheral blood

There is no evidence of increased frequency of B cells in peripheral blood of MG patients. However, circulating B cells of these patients, particularly when positive for anti-AChR antibodies, display increased expression levels of activation markers (e.g. CD23, CD71) (19, 42), as found for thymic B cells. In line with this observation, peripheral blood mononuclear cells (PBMCs) from MG patients have enhanced ability to secrete IgG, and anti-AChR antibody secretion correlates with IgG secretion (43). The levels of several cytokines involved in B cell activation and antibody production are increased in serum of MG patients, and positive correlations were found between IgG and interleukin (IL)-6 levels (19, 43). Among B cell-stimulating cytokines, higher levels of serum BAFF (44), and higher frequency of circulating B cells expressing BAFF receptor (BAFF-R), have been reported in MG patients, in both AChR- and MuSK-MG (45, 46), indicating abnormal B cell survival. Regulatory B cells producing IL-10 (B10 cells), which play an important immunosuppressive role through potent inhibition of B- and T-cell responses, were found to be reduced in peripheral blood of both AChR-MG and MuSK-MG patients, and their reduction was associated with disease severity (46, 47). This finding represents an additional MG-associated B cell defect implicated in immune tolerance breakdown. By deep sequencing, large-scale abnormalities were found in both the naïve and memory B cell receptor repertoires of AChR- and MuSK-MG patients, indicating disturbed B cell repertoire as a fundamental MG component with distinct properties between the two disease subgroups (48). Interestingly, the naïve B cell repertoire of MuSK-MG patients includes self-reactive clones capable of specific binding to MuSK with high affinity, suggesting that the MuSK antigen might trigger B cell activation and differentiation toward memory B cells and antibody-secreting cells that directly contribute to the disease (49).

AChR-MG patients are characterized by enhanced frequency of circulating plasma cells, which account for autoantibody production (50). Of note, anti-AChR antibody-producing plasma cells have been identified in the bone marrow (51), a well-recognized niche for long-lived plasma cells that are able to survive for prolonged periods of time and maintain long-term humoral immunity. Longevity and radio-resistance are typical features of persisting anti-AChR antibody-producing plasma cells (52). Since EBV-infected B cells are long-lived cells, presence of long-lived plasma cells in MG patients is in line with a possible contribution of EBV to chronic autoimmunity in MG. In contrast, short-lived plasmablasts are key autoantibody producers in MuSK-MG patients (53, 54), thus explaining the favorable outcome of B cell-depleting therapies in these patients compared to those positive for anti-AChR antibodies, as discussed below.

### 3.2 Targeting B-cell compartment as strategy “to strike at the heart of autoimmunity”

Due to their essential role in the autoimmune response, B cells are the main candidate target cells for effective therapies in MG to inhibit autoimmunity. Biological drugs targeting B cells can be categorized into drugs that directly target B or plasma cells, drugs that block the survival and differentiation of B cells, and drugs targeting cytokines involved in the differentiation and activation (**Figure 1**).

#### 3.2.1 Drugs directly targeting B cells

Rituximab (RTX), a biological drug that directly and specifically targets B cells, has been approved in several B cell-mediated autoimmune conditions, such as RA and SLE (55–57). RTX is a chimeric murine-human IgG1 monoclonal antibody that depletes all B cell subsets (but not pro-B cells, plasmablasts and long-lived plasma cells) through specific binding to the transmembrane CD20 antigen and induction of apoptosis of the target cell, antibody-dependent cell-mediated cytotoxicity, or complement-dependent cytotoxicity (55).

RTX effectiveness in AChR-MG patients has been demonstrated in several uncontrolled studies and case reports (58), but a multicenter, placebo-controlled, randomized phase 2 trial (BeatMG Study) failed to achieve the primary steroid-sparing outcome (60% with RTX versus 56% with placebo), as well as secondary outcomes, in patients with mild-to-moderate gMG (**Table 1**) (59). Low disease activity, concomitant prednisone in a dose range of 15–60 mg/d and a consistent placebo response likely affected the negative results of this trial. Successful B-cell depletion was achieved in the treatment arm, but the drug did not affect significantly anti-AChR antibody levels (59). Contrariwise, RTX has been proven to almost completely eliminate autoantibodies in MuSK-MG patients in line with long-lasting disease improvement (60) (**Table 1**). A systematic review of case reports and series from Tandan and colleagues (61) showed that MuSK-MG patients are more responsive to RTX than AChR-positive, showing markedly decreased post-treatment antibody levels, as early as 3 months after RTX-mediated B cell depletion, and a longer clinical improvement in most patients. The reason for the different response to RTX between AChR- and MuSK-MG patients is likely related to the different type of autoantibody-producing cells in the two disease subgroups: as reported above, autoantibodies against MuSK are produced by short-lived plasmablasts that are continuously regenerated from autoreactive CD20-positive B cells, thus depletion of these cells may be effective; autoantibodies against AChR likely derive from long-lived plasma cells, and hence total circulating IgGs remain constant in the long-term after CD20 depletion therapy (53, 54, 62).

Recently, RTX was found to increase the probability of achieving minimal disease manifestations despite low doses of corticosteroids in the short to medium term in new-onset AChR-MG patients with generalized disease (RINOMAX study), likely because early immune response may derive from plasmablasts and short-lived plasma cells, thus suggesting the use of RTX early in the disease course to reduce the risk of worsening or the need for additional therapies (63) (**Table 1**). However, the RINOMAX study lasted 48 weeks and the long-term benefit-risk balance was not addressed. Nevertheless, duration of RTX beneficial effects may be limited both in AChR- and MuSK-MG patients, since disease relapse can occur in patients who achieved drug-induced remission (54). This is likely due to resurgence of pathogenic B cells, highlighting the need for retreatment (64). Repopulation of pathogenic versus regulatory B cells is an important factor underlying clinical response to RTX. Indeed, it has been demonstrated that MG patients who respond well to the drug exhibit rapid repopulation of B10 cells compared to non-responder patients, who show a delayed B10 cell repopulation (65). A new trial on RTX efficacy in AChR-MG is ongoing (REFINE, https://clinicaltrials.gov/study/NCT05868837) and it is expected to be completed in 2025 (**Table 1**). In addition, a phase 3 clinical trial on Inebilizumab, a humanized antibody that binds to the B cell-specific antigen CD19, is ongoing (MINT, https://clinicaltrials.gov/study/NCT04524273) and should be completed in 2029 (**Table 1**). Inebilizumab looks promising for both AChR- and MuSK-MG as CD19 expression, compared to CD20, is maintained in plasmablasts and plasma cells, thus the drug is able to deplete also these pathogenic cells in MG patients.

Since B cells express Bruton’s tyrosine kinase (BTK), an enzyme crucial for B-cell activation, growth and differentiation, another promising anti-B cell therapy could be based on BTK inhibitors. These drugs were found to be beneficial in treating some autoimmune diseases, such as SLE and RA (66), but clinical studied are needed to evaluate their efficacy MG. Several clinical trials are also ongoing in MS (67).

#### 3.2.2 Drugs directly targeting plasma cells

Therapies specifically targeting plasma cells might represent an appropriate therapeutic approach for AChR-MG. Bortezomib, a small-molecule proteasome inhibitor approved for the treatment of multiple myeloma and mantle cell lymphoma, depletes plasma cells and blocks specific autoantibody production in primary thymic cell cultures of EOMG patients (68). In the experimental autoimmune MG model (EAMG), the drug was effective in reducing anti-AChR antibody titers and prevent immune-mediated destruction of the neuromuscular junction (68). A phase 2 pilot study (TAVAB) on bortezomib in treatment-refractory patients with autoimmune diseases, including patients with AChR-MG, SLE and RA (n=6 for each disease) has been carried out, but the results have not been published yet (69) (**Table 1**).

Another drug directed against plasma cells is TAK-079, a high-affinity antibody specific for CD38, that is expressed on plasmablasts, plasma cells, but also natural killer cells and other non-immune cells (70) (**Table 1**). The drug has been evaluated in a phase 2 trial for AChR- and MuSK-MG (https://clinicaltrials.gov/study/NCT04159805) but no publication on this study is yet available.

Recently, chimeric antigen receptor (CAR) T cells engineered with RNA (rCAR-T) were developed to target the B-cell maturation antigen (BCMA) expressed on plasma cells (71). CAR molecules brings the extracellular target binding domain of an antibody directed toward a specific target together with the intracellular T-cell activation protein domains, enabling T-cell activation upon contact with the target antigen without antigen presentation by professional cells and regulatory checkpoints (72). Compared to cells engineered with the conventional DNA approach, in which CAR-expressing DNA is integrated permanently into the T-cell genome and replicates with each cell division, CAR-encoding mRNA does not replicate together with the activated and proliferating rCAR T cells, thus making rCAR-T safer than classical CAR-T therapy (71). A prospective open-label (OLE) phase 1b/2a study (MG-001) on the efficacy of Descartes-08, an autologous CD8+ T-cell product transfected with RNA to express the anti-BCMA targeting CAR protein, was carried out in seropositive (AChR-, MuSK- or LRP4-positive) gMG patients (n=14) by Granit and colleagues (71). They demonstrated that anti-BCMA rCAR-T therapy was safe and able to meaningfully decrease MG severity scales (i.e. MG-ADL, MGC, QMG, and MG-QoL-15r scores) at up to 9 months of follow-up (71). A more complete assessment of Descartes-08 efficacy is ongoing in a placebo-controlled study in gMG patients (https://www.clinicaltrials.gov/study/NCT04146051).

#### 3.2.3 Drugs blocking survival of B cells

B cell targeting drugs can exert an indirect effect, by promoting inhibition of B cells via blockage of their stimulating factors, such as BAFF that is able to induce B-cell survival and differentiation, the levels of which are increased in MG patients’ sera (44).

Belimumab is a human IgG1 monoclonal antibody that blocks both soluble and membrane-bound BAFF, thus inhibiting its interaction with the cognate receptors BAFF-R, B-cell maturation antigen (BCMA), and transmembrane activator and CAML interactor (TACI) (73). The drug has been approved for the treatment of SLE and is promising for the treatment of patients with RA and Sjögren’s syndrome (73). Conversely, a phase 2, double-blind, multicenter randomized trial carried out on gMG patients, who were all AChR-MG except for two MuSK-MG patients, showed no statistically significant differences in the primary endpoint (i.e. mean change in the Quantitative Myasthenia Gravis, QMG, score) between placebo and active treatment after 24 weeks (74) (**Table 1**). The trial failure could be explained by the inclusion of a small number of patients (n=40), who had mild-moderate disease, as well as by the presence of long-lived autoantibody-producing plasma cells in AChR-MG patients. No further trial on belimumab efficacy has been conducted in MG.

Telitacicept is another drug providing an option to block B cells in patients with autoimmune diseases by acting on B cell survival factors. It is a fusion protein binding the TACI receptor to inhibit both BAFF and APRIL, another cytokine sustaining B-cell survival (75). A clinical trial (https://clinicaltrials.gov/study/NCT05737160) on Telitacicept safety and efficacy in gMG patients is open and is expected to be completed in 2027.

Inflammatory cytokines play a key role in activation and differentiation of immune system cells, including B cells. Thus, therapies targeting these proteins can indirectly affect B cell populations. IL-6 has been implicated in autoantibody production, MG activity and severity (76–78), thus representing another therapeutic target. Satralizumab is a humanized IL-6 receptor monoclonal antibody developed to provide durable suppression of IL-6 signaling. The LUMINESCE phase 3 randomized, double-blind, placebo-controlled study evaluated satralizumab efficacy in MG patients (https://clinicaltrials.gov/study/NCT04963270) (**Table 1**). Only a modest improvement in MG-ADL and QMG scores from baseline to week 24 in AChR-MG patients has been reported in the abstract from: https://medically.roche.com/global/en/neuroscience/aan-2024/medical-material/AAN-2024-presentation-habib-primary-and-secondary-results-of-LUMINESCE-pdf.html (access on 13^th^ may 2024).

#### 3.2.4 Other strategies to deplete autoreactive cells

Transplantation with autologous hematopoietic stem cells (HSCT) represents a strategy to eliminate autoreactive immune system cells, including B cells. This procedure, preceded by immunoablative high-dose chemotherapy, has been evaluated as a potential intervention to restore self-tolerance in patients with refractory and severe MG (79). The treatment was found to lead to prolonged (29–149 months) and complete symptom- and treatment-free (CSR) remission in 7 MG patients (i.e. six AChR-positive; one negative for anti-AChR antibodies with unknown anti-MuSK and -LRP4 autoantibody status) (79). In this very small patients’ cohort, HSCT was tolerable, and acute toxic effects were not observed. However, since the immunoablative conditioning regimen may cause important short-term complications, including opportunistic infections and rarer cardiac, renal, or other organ toxic effects, as well as late complications, such as endocrine dysfunction, it should be carefully explored in selected patients in whom the risks of treatment are outweighed by potential benefits.

## 4 Antibody recycling by FcRn as physiological mechanism prolonging autoantibody effects

Current therapies for MG suppress the immune system without specifically targeting the autoantibodies, which play a central role in the disease development. Thus, biological drugs able to reduce autoantibody levels are promising to counteract MG, particularly AChR-MG for which anti-B cell drugs have limited efficacy due to the action of autoantibody-producing long-lived plasma cells. IgG have a longer half-life compared to other immunoglobulin classes (i.e. over 3 weeks *versus* 5–7 days) (80). Drugs promoting their clearance, or able to reduce their persistence in the body, represent an ideal strategy to treat MG. A key molecule ensuring prolonged half-life of IgG is the neonatal Fc receptor (FcRn): its biological function is to maintain IgG concentration in the circulation and interstitial fluids (80). In the era of biologicals, this function has offered a therapeutic potential, leading to the development of innovative drugs targeting this receptor to lower circulating IgGs in antibody-mediated autoimmune conditions, such as MG (**Figure 1**).

### 4.1 FcRn-IgG immunobiology

FcRn belongs to the heterogeneous family of MHC molecules, from which it differs for limited diversity and inability to present antigens. FcRn is a beta-2 macroglobulin-associated protein expressed in a wide variety of tissues, including epithelia, endothelia, intestinal cells, kidney, liver, and placenta (80). FcRn is critically implicated in the materno-fetal IgG transfer, a mechanism that protects offspring from infections in early life. The receptor continues to play a key immunological role beyond the neonatal period, being able to protect IgG from intracellular catabolism and recycle them back into the circulation. Intracellularly, the FcRn binds the Fc portion of IgG with high affinity within the pH acid microenvironment typical of endosomes, inhibits IgG lysosomal degradation and promotes their release outside the cells into the neutral pH circulation milieu (80, 81). This process, mainly occurring in the vascular endothelium, increases the IgG half-life, according to the specific binding affinity of different IgG isotypes to FcRn. IgG3 have been proven to have the lowest binding potential to the receptor, and indeed they have the lowest half-life in the circulation compared to other isotypes, likely as an effect of competition with IgG1 for FcRn (82).

FcRn is also widely expressed on the cell surface of several hematopoietic cells, including monocytes, macrophages, dendritic cells (DCs), neutrophils and B cells (83). Thus, the FcRn is involved in additional immune functions, other than IgG transport and recycling, including potentiation of innate immune responses to IgG forming immune complexes with their antigens, that is important for immune surveillance against infections (81). In neutrophils, the receptor enhances phagocytosis of IgG-opsonized bacteria (84). In DCs and other antigen presenting cells, the FcRn participates in the presentation/cross-presentation of antigenic peptides by MHC class II and I to CD4+ and CD8+ T cells, by directing the internalization, trafficking and processing of antigen-bound IgG immune complexes (81, 84). In this way, the receptor contributes to the activation of adaptive immune responses. It has been demonstrated that production of pro-inflammatory cytokines (e.g., IL-6, TNF- α) by innate immune cells in response to IgG immune complexes strictly depends on FcRn, since it requires IgG binding to FcRn, and can be inhibited by FcRn antagonists (85). Thereby, pharmacological FcRn inhibition can result in an anti-inflammatory action via mitigation of pathogenic inflammatory responses in the context of inflammatory and autoimmune conditions.

FcRn expression is modulated in different ways depending on the different immunological microenvironments: the exposure of human PBMCs to TNF-α, LPS or CpG is able to induce a significant increase of FcRn expression via NF-κB signaling pathways; contrariwise, activation of the JAK/STAT-1 signaling pathway by IFN-γ can down-regulate functional FcRn expression (86, 87). In addition, the promoter region of the FcRn-encoding gene, *FCGRT*, contains variable number of tandem repeats (VNTRs) that affect the receptor expression: subjects homozygous for VNTR3, which is the most common variant (~84% of human population), have higher FcRn expression, and increased IgG binding ability, than heterozigous subjects bearing the VNTR2 variant (VNTR2/VNTR3), which is the second most frequent variant (~13% of the human population) followed by VNTR1,4 and 5 (~3% of the human population) (88). These observations implies that the function of FcRn, and the magnitude of beneficial effects of its therapeutic blockage, could vary in different disease contexts and genetic backgrounds.

### 4.2 FcRn blockade in MG

The role of the FcRn as regulator of IgG homeostasis has made it an attractive target for precision medicine in MG to reduce pathogenic antibody levels in a specific manner compared to plasmapheresis or immunoglobulins.

Preclinical studies showed safety and efficacy of an anti-FcRn monoclonal antibody, 1G3, in a passive and active model of EAMG: in rats in which MG was induced by passive transfer of anti-AChR antibodies, 1G3 treatment resulted in dose-dependent improvement of symptoms and reduction of pathogenic antibody levels in serum; in rats immunized with the AChR, the treatment significantly reduced the severity of the disease symptoms and the levels of both total IgG and anti-AChR antibodies (89). Similarly, in a mouse model of MuSK-MG, obtained by injections with purified MuSK-MG patients’ IgG4, FcRn blockade by efgartigimod reduced IgG4 levels and determined significant *in vivo* muscle function improvements, thus highlighting the potential of FcRn-targeted therapies to effectively improve MG (90).

Efgartigimod (ARGX-113) is the first FcRn antagonist approved (December 2021) in the USA by the US Food and Drug Administration (FDA). It is a humanized IgG1-derived Fc fragment able to bind FcRn with high affinity, thus competing with endogenous IgG and lowering their levels (91).

The phase 3 double-blind, multicentric randomized-controlled ADAPT study assessed the efficacy and safety of efgartigimod as add-on therapy in 167 patients with gMG on a stable dose of at least one IS treatment, including 129 (77%) AChR-MG patients, 6 (4%) MuSK-MG patients and 32 double (AChR/MuSK-) negative patients (92) (**Table 2**). Efgartigimod (10 mg/kg) or matching placebo was administered as four infusions per cycle (one infusion per week), repeated as needed depending on clinical response no sooner than 8 weeks after initiation of the previous cycle. Both in the AChR-positive and -negative patients, IgG levels significantly decreased with each cycle. A significantly higher proportion of AChR-MG patients in the drug group were MG-Activities of Daily Living (MG-ADL) responders (68%) than in the placebo group (30%) after the first cycle. Moreover, in cycle 1 the drug led to sustained improvements of the total mean scores for MG-ADL, QMG, MG Composite (MGC), and MG Quality of Life 15-item Scale - Revised (MG-QoL15r) rating scales (92). In patients who received a second cycle, a greater proportion of patients in the drug group (71%) were MG-ADL responders compared with the placebo group (26%), with similar rates observed after cycle 1. The drug was well tolerated and the safety profile was good. The results in the AChR-negative patients were similar to those in the AChR-MG population. Efgartigimod effectiveness in inducing clinically meaningful improvement in MG-ADL was recently reported by Katyal and colleagues (93), who analyzed the treatment outcomes in a cohort of 37 AChR-MG patients, with all except one having completed at least one cycle and 28 patients having completed at least two cycles. Clinically meaningful improvement in MG-ADL was achieved in 72% (26/36) of patients after the first cycle, including 3 of 4 patients with thymoma, and 25% (7/28) of patients achieved minimal symptom expression status after the second cycle (93) (**Table 2**). The long-term effect of efgartigimod along 14 months, and its impact on the disease course, was more recently reported in 19 MG patients (AChR-, MuSK-, LRP4-positive or triple negative MG). During the year before treatment 8 of 19 patients (42%) were hospitalized, and 15 of 19 (79%) needed treatment with plasma exchange or immunoglobulins; three of 19 (16%) were admitted to the intensive care unit. During efgartigimod, none of the patients was hospitalized and only one patient required plasma exchange and intravenous immunoglobulins (94) (**Table 2**). Positive results on the real world use of efgartigimod in 36 seropositive and seronegative MG have been recently reported from Japan (95) (**Table 2**). Long-term efgartigimod safety, tolerability, and efficacy has been confirmed by the OLE (up to 3-year extension) study of the ADAPT (96). Another FcRn blocker is Rozanolixizumab (UCB7665), a human IgG4 monoclonal antibody targeting the FcRn IgG binding region, whose safety and efficacy in gMG has been evaluated in a randomized, double-blind, placebo-controlled, adaptive phase 3 study (MycarinG) (97) (**Table 2**). The study included 200 gMG patients, who received subcutaneous infusions once a week for 6 weeks of either rozanolixizumab 7 mg/kg (n=66), rozanolixizumab 10 mg/kg (n=67), or placebo (n=67). Both AChR- (n=179) and MuSK-positive patients (n=21), under conventional IS treatment, were included. Significant MG-ADL reductions from baseline were observed at day 43 (primary efficacy endpoint) for both the two rozanolixizumab groups compared to the placebo group. Improvements in MG-ADL, MGC, QMG, and Myasthenia Gravis Symptoms PRO (98) scores were observed as early as day 8 from the treatment start, the first time-point at which efficacy was assessed, and returned towards baseline levels by day 99, according with rapid reductions in total IgG at day 8 and their gradual increase by day 99. MuSK-MG patients had greater score reductions than the overall population, and all of them achieved an MG-ADL response (97). The MycarinG study results thus provided further support to safety and efficacy of FcRn inhibition for the treatment of gMG.

Nipocalimab (M281) is a fully human IgG1 monoclonal antibody with high affinity for the IgG binding site on FcRn. A phase 2 multicenter, randomized, double-blinded placebo trial (Vivacity-MG) assessed the nipocalimab efficacy in moderate-to-severe gMG (99) (**Table 2**). The study included 68 MG patients randomly assigned to receive different intravenous doses of the drug (4 treatment groups) or placebo for 8 weeks. There were no treatment discontinuations due to side effects, and no difference between the treatment and placebo arms for adverse events (i.e. infections, headaches). A greater proportion of nipocalimab-treated patients exhibited rapid improvement (within two weeks of treatment) in MG‐ADL across all the 4 dosing arms compared to the placebo arm. The drug led to substantial reductions in serum total IgG and anti‐AChR autoantibodies that significantly correlated with MG‐ADL improvement (99). Safety and efficacy of nipocalimab is under investigation in an ongoing phase 3 multicenter study in adult patients with seropositive gMG (https://clinicaltrials.gov/study/NCT04951622).

Recently, Nowak and colleagues published the results of a Phase 2 proof-of-concept, randomized, double-blind, placebo-controlled trial with an OLE, on the efficacy of batoclimab, a subcutaneous fully human anti-FcRn monoclonal antibody, in AChR-positive gMG patients (100) (**Table 2**). The drug was associated with significantly greater reductions in total IgG and anti-AChR antibodies from baseline to 6 weeks than placebo. MG-ADL, QMG, MGC, and MG-QoL15r scores showed improvements over time, but differences between the two treatment arms did not reach statistical significance, likely due to the small sample size (n=17) (100). A Phase 3 study performed in a higher number (n=132) of patients with gMG, including both AChR- and MuSK-positive patients (101), showed batoclimab ability to significantly increase the rate of sustained MG-ADL improvement, as early as week 2 for 4 or more consecutive weeks, with clinical effects and IgG reduction being similar to those previously reported for efgartigimod and rozanolixizumab. Currently, batoclimab therapeutic efficacy is under evaluation in another Phase 3 trial in AChR-positive gMG patients, expected to be completed in 2025 (https://clinicaltrials.gov/study/NCT05403541).

## 5 Complement system as key damage mediator and therapeutic target in AChR-MG

The complement system is the main mediator of the NMJ damage caused by anti-AChR autoantibodies (102), thus representing an ideal target of precision medicine therapies to specifically and effectively treat AChR-MG (**Figure 1**).

### 5.1 Role of complement system in AChR-MG

Anti-AChR autoantibodies belong to the IgG1 and IgG3 subclasses, that are the strongest activators of the complement system. Indeed, pathogenicity of these autoantibodies, but not that of anti-MuSK antibodies that belong to IgG4, is mainly due to complement fixation at the NMJ ultimately leading to postsynaptic membrane destruction and impairment of the neuromuscular transmission (102–104). Additional pathogenic mechanisms of anti-AChR antibodies are the direct functional block of the receptor and antigenic modulation, the last consisting in the cross-linking of two adjacent AChR molecules by the same antibody, causing AChR endocytosis and degradation (104).

The complement system, consisting of nearly 50 blood proteins, plays a key role in acute inflammation and defense against common pathogens as part of the innate immune response. Complement activation generates a robust proteolytic cascade that leads to opsonization for phagocytosis and osmotic/colloidal lysis of the pathogen, as well as generation of a potent inflammatory response through the production of pro-inflammatory molecules. Furthermore, the complement system acts to remove antigen-antibody complexes from circulation and is involved in adaptive immune responses, participating in the regulation of T and B cell activation (105).

In MG, complement-mediated damage of the NMJ occurs via the classical complement pathway, which starts with C1q binding to the Fc domain of the autoantibodies. Critical steps of the complement cascade are then the cleavage of C3 into C3a and C3b, and of C5 in C5a and C5b, the latter leading to the formation of the membrane attack complex (MAC, C5bC6C7C8C9 also known as C5b-9) that causes focal lysis of the post-synaptic membrane, disruption of post-junctional folds, and ultimately reduction of functional AChRs (102, 106, 107).

Several preclinical studies have demonstrated the role of the complement system in EAMG, as well as the efficacy of complement inhibition via recombinant proteins, chemicals, monoclonal antibodies and small interfering RNA (siRNA), in improving MG symptoms (108–113).

In muscle biopsies from MG patients, IgG and C3 deposition was localized at identical sites of post-synaptic membrane, and specifically on debris of junctional folds in the synaptic clefts and disintegrating junctional folds (114). Deposition of C9, the main component of the final and stable MAC, was also observed at the MG end plate regions, with the most intense depositions associated with the destroyed NMJs, indicating that NMJ loss may be largely due to complement activation (114).

Complement regulators are present on host cell surfaces to prevent autologous destruction by the complement system. Mice deficient for both the decay accelerating factor 1 (DAF1 or CD55), which inhibits C3 and C5 convertases, and the MAC inhibitory protein (MAC-IP or CD59), which inhibits MAC formation, developed a significantly worse EAMG associated with crisis, further supporting the role of complement role in MG (115).

Recently, Obaid and colleagues developed an assay based on the EK293T cell line modified to disrupt complement regulator gene expression via CRISPR/Cas9 genome editing, that was used to measure patient-specific autoantibody-mediated MAC formation by flow cytometry (116). By means of this system, they observed a subset of AChR-MG patients with high complement activity, who are likely patients in whom complement activation is the major disease mechanism, whereas the remaining patients showed low activity, reflecting possible complement-independent autoantibody-mediated mechanisms of NMJ damage (116). Circulating complement protein profile may be used as a biomarker for evaluating patient-specific complement activation: (i) Romi and colleagues found reduced C3 and C4 serum levels in AChR-MG patients with high autoantibody titers, indicating increased *in vivo* complement consumption (117); (ii) Liu and colleagues showed lower C3 levels in serum of AChR-positive compared to AChR-negative gMG patients and healthy controls (118), again suggesting complement activation-dependent C3 consumption; (iii) C2 and C5 levels were significantly reduced, and C3, C3b and C5a increased, in plasma of AChR-MG patients compared to controls in our recent study, suggesting “C2, C3, C5, C3b, C5a” as a profile suggesting complement activation (119); iv) increased levels of complement activation products (e.g. C5a, C3a, SC5b-9, C4a) were found in AChR-MG compared to controls by Staschei and colleagues (120), and v) lower serum C5 levels, indicating consumption, associated with higher sC5b-9 levels, were found in AChR-MG compared to patients with non-inflammatory neurological disorders by Ozawa and colleagues, further indicating complement activation in AChR-MG (121). Discrepancy among the results from the different studies could be due to differences in the clinical features of the patients’ cohorts (e.g. disease severity; IS treatments) or to technical issues related to the different methods used for the complement protein dosage, as well as to sample handling and storage and sample types (serum versus plasma). Recommendations for complement laboratory analyses should indeed be rigorously followed to avoid biased results, due to *in vitro* complement activation and consumption (122).

### 5.2 Complement inhibitors to block neuromuscular junction damage by anti-AChR-antibodies

Since complement activation results from a cascade of multi-step events, many potential targets are available as candidates for therapeutic intervention.

C5 is a central component of the complement cascade as its cleavage into C5a and C5b is critical for MAC formation that is initiated by C5b (106). Biological drugs targeting C5 have emerged as a successful strategy to treat complement-mediated diseases, including MG (123).

Eculizumab is a fully humanized monoclonal antibody binding C5, approved by FDA for the treatment of AChR-MG patients with gMG (124). By inhibiting C5 cleavage into C5a and C5b, the drug is able to block the MAC formation and abrogate complement-mediated damage. Preclinical studies showed that administration of an anti-C5 monoclonal antibody in a passive immunization rat model not only restored strength in the animals, but also inhibited deposition of C9, thereby preventing endplate disruption (110). Eculizumab efficacy and safety in refractory AChR-positive gMG patients were demonstrated in the phase 3 REGAIN trial, and the following OLE study, demonstrating that eculizumab is able to elicit a rapid (i.e. by weak 1) and sustained (i.e. at least 130 weeks) improvement in muscle strength across ocular, bulbar, respiratory, and limb muscle groups and in daily activities (125–127) (**Table 3**).

Ravulizumab is another FDA-approved human anti-C5 monoclonal antibody with similar mechanism of action of eculizumab but with a longer half-life, requiring less frequent infusions (every 8 weeks compared to 2 weeks for eculizumab). The phase 3 CHAMPION-MG study showed the drug ability to induce a clinically meaningful response in terms of significant MG-ADL and QMG reduction in AChR-MG gMG patients, with a rapid improvement within 1 week of treatment initiation in the treatment versus the placebo group, and adverse events rates were comparable between the two groups (128). The OLE phase of this study showed a sustained efficacy and long-term safety of ravulizumab, administered every 8 weeks, with rapid improvements in MG-ADL and QMG scores (within 2 weeks) and maintained up to 60 weeks (129) (**Table 3**).

Zilucoplan is a macrocyclic peptide able to allosterically inhibit C5 cleavage. Compared to eculizumab and ravulizumb, that are administered intravenously, Zilucoplan offers the advantage to be administered subcutaneously at-home once daily by self-injections. Safety and efficacy of zilucoplan has been proven in patients with moderate to severe gMG in the phase III RAISE trial (130) (**Table 3**). Indeed, MG patients assigned to zilucoplan showed a greater reduction in MG-ADL score from baseline to week 12, compared with those assigned to placebo, with incidences of serious adverse events and infections similar in both groups. An OLE study is ongoing (RAISE-XT, https://clinicaltrials.gov/study/NCT04225871).

Other drugs developed to block C5 for treatment of complement-mediated disorders are pozelimab and cemdisiran. The first is a fully human IgG4 monoclonal antibody against C5 with a proline substitution to promote stabilization of the disulfide bonds between the two heavy chains (IgG4P); the second is a small interfering N-acetylgalactosamine-conjugated ribonucleic acid (siRNA) which interferes with mRNA for C5, thus offering the opportunity to reduce C5 hepatic synthesis and hence circulating levels. The combination of pozelimab and cemdisiran was recently found to inhibit complement activity more efficiently (i.e. durable and more complete suppression) than the monotherapy with either pozelimab or cemdisiran in non-human primates, thus opening the way to clinical investigations on the potential of long-acting treatment with these two drugs for complement-mediated disease treatment (131). A phase 3 trial on the efficacy of a combination therapy based on intravenous pozelimab and subcutaneous cemdisiran is ongoing in gMG (NIMBLE, https://clinicaltrials.gov/study/NCT05070858).

Along with C5, additional complement cascade components are promising targets to counteract AChR-MG. Danicopan (ALXN2040) is an oral small molecule complement factor D (FD) inhibitor that showed improved benefit-risk profile as add-on therapy to ravulizumab or eculizumab in patients with complement-mediated paroxysmal nocturnal hemoglobinuria (PNH) compared to placebo (132). A phase 2 study on vemircopan, a similar oral factor D inhibitor molecule (ALXN2050) qualified for monotherapy in PNH, is ongoing in gMG patients (https://clinicaltrials.gov/search?intr=NCT05218096) (**Table 3**). Since FD is essential for complement activation being necessary for the formation of the C3 convertase (107), its blockage is a promising approach to counteract the complement-mediated damage of autoantibodies and hence to treat AChR-MG.

Another oral inhibitor of complement activity is Iptacopan (LNP023), a small-molecule factor B inhibitor approved for PNH. The drug is able to block the activation of the alternative pathway at the level of the C3 convertase; indeed, factor B is a serine protease that drives the central amplification loop of this pathway (133). Inhibition of factor B has been proven to prevent complement activation in different complement-dependent diseases, thus sounding promising also for the treatment of MG.

## 6 Future research directions to achieve precision medicine in MG

MG is a chronic autoimmune disease characterized by debilitating symptoms and unpredictable course. The development of biological drugs and the demonstration of their efficacy in clinical studies promise to profoundly change the treatment scenario for MG. Compared to conventional IS drugs, biological drugs are able to target specific disease effector molecules, thus minimizing the risk of adverse events. However, these drugs are used as add-on therapy, and hence their effect in monotherapy and in the early disease phase is unknown. Moreover, their high costs may limit their prescription as first-line therapy. As for IS drugs, response to biological drugs may vary among patients, according with individual differences in genetic, epigenetic, environmental and lifestyle factors and likely in the pathophysiological mechanisms underlying the disease.

Heterogeneity in MG clinical manifestations and inter-individual variation in the response to treatment highlight the need to adopt safer, more tailored and effective precision medicine approaches, still lacking in MG. The availability of different biological drugs highlights the importance of establishing criteria to select the better treatment option for individual patients or patients’ subgroups. The identification of genetic, epigenetic or protein biomarkers able to predict unresponsiveness to conventional IS drugs and clinical benefits of the different available biologicals is the key to individualization of MG care. When identified and properly validated, biomarkers of response to biological drugs may greatly facilitate introduction of these drugs in clinical practice, thus promising to improve therapeutic success in a cost-effective manner. Research on biomarkers and biomarker entry into clinical trials are pivotal steps for precision medicine in MG.

Research on biomarker identification may identify novel mechanisms associated with distinct pathological phenotypes, opening new hypotheses on MG pathogenesis and treatment.

An open research area is that of B10 cells. Therapeutic approaches aimed at restoring immunosuppressive B10 cell frequency could be successful for re-establishing B cell tolerance and reducing antibody-producing plasma cells. Moreover, since AChR-MG-associated long-lived plasma cells are resistant to most IS treatments and B cell-targeted therapies, they represent promising targets for therapies aimed at reducing autoantibody production in AChR-MG. Antigen-specific B-cell depletion has been recently described as a promising strategy for MuSK-MG patients (134). T cells expressing a MuSK chimeric autoantibody receptor with CD137-CD3ζ signaling domains (MuSK-CAART) were engineered to target B cells expressing anti-MuSK autoantibodies. In an experimental autoimmune MG mouse model, MuSK-CAART reduced anti-MuSK IgG but not B cell counts or total IgG levels, indicating that MuSK-specific B cell depletion occurred in treated mice. Off-target cytoxic interactions were not identified in mouse tissues or using high-throughput human membrane proteome array, thus suggesting MuSK-CAART as a safe and efficient strategy for the treatment of MuSK-MG (134).

FcRn blockage is particularly efficient in reducing circulating IgG and specific autoantibodies. In-depth investigation into the mechanisms underlying FcRn regulation of immune system cell (e.g. antigen presenting cell) functions could be useful to understand additional biological effects of drugs targeting this receptor, helpful for biomarker identification.

The involvement of the complement system has been widely demonstrated in AChR-MG patients and animal models. To date, it is unknown whether the prevailing autoantibody effector mechanism (e.g. complement-mediated damage vs antigenic modulation) is different among different MG patients, or whether different mechanisms co-exist in the same patient. As described above, by using a complement regulator-lacking HEK293T cell line-based model Obaidi and colleagues (116) measured the anti-AChR autoantibody-mediated MAC formation through flow cytometry in AChR-MG patients with a wide range in terms of disease severity and autoantibody titer. They identified a subset of patients lacking association between MAC formation and autoantibody binding or disease severity, indicating other complement-independent autoantibody-mediated mechanisms impairing the NMJ (116). The remaining patients showing a relationship between MAC formation, autoantibody binding and disease severity represent patients expected to benefit from complement inhibitor therapy (116). This and similar *in vitro* models able to predict the autoantibody-mediated effector mechanism in individual patients could be useful to pre-select AChR-MG patients as candidates for anti-complement therapies. These patients could also be identified via complement-related protein profiling in serum, since complement components and activation products (119), or other proteins markers of the patient-specific degree of complement activity, could be easy-to-use biomarkers to predict responsiveness to anti-complement drugs. Common inherited variants in genes encoding complement components and regulators have been associated with complement-related diseases, suggesting that they can affect complement activity (135). These variants (i.e. complotype) may contribute to the individual susceptibility to a higher or lower complement activation degree, thus representing additional candidate predictive biomarkers to be explored for their association with patient-specific response to anti-complement drugs and hence useful for treatment tailoring in each patient.

## 7 Conclusions

The advances in biological drugs’ research promise to significantly change the therapeutic scenario for MG, since these drugs have the potential to overcome the limitations of conventional non-specific IS therapies and greatly increase therapeutic success. Different drugs have been developed, which specifically target different and distinct immune cells or mechanisms (**Figure 1**), thus highlighting the need to identify biomarkers (i.e. clinical, pathophysiological, molecular) of response predictive of the best option in individual patients. Despite stunning progresses in OMICs technology, the identification of OMIC-based biomarkers is still an open research area, and a top priority for research in MG. Biomarker profiling is indeed pivotal for patients’ stratification in endotypes, offering the opportunity, or challenge, to discover endotype-associated pathogenetic features, factors and mechanisms to solve MG complexity and guide clinical trial design and therapy. In our vision, targeted therapies based on biological drugs and guided by biomarkers represent the new frontier for MG care to achieve precision medicine and improve patients’ health and quality of life.

## Author contributions

PC: Writing – original draft. RM: Writing – review & editing. CA: Writing – review & editing.
